# Timelines to initiate a phase III trial across the globe: a sub-analysis of the APHINITY trial

**DOI:** 10.3332/ecancer.2022.1379

**Published:** 2022-04-29

**Authors:** Maria Alice Franzoi, Marion Procter, Chris Twelves, Noam Ponde, Daniel Eiger, Orianne Emond, Emma Clark, Damien Parlier, Sébastien Guillaume, Linda Reaby, Evandro de Azambuja, Jose Bines

**Affiliations:** 1 Clinical Trials Support Unit, Jules Bordet Institute, Brussels, Belgium; 2 Frontier Science Scotland Ltd, Kincraig, Kingussie, UK; 3 University of Leeds and Leeds Teaching Hospitals Trust, Leeds, UK; 4 AC Camargo Cancer Center, São Paulo, Brazil; 5 F Hoffmann-La Roche Ltd, Basel, Switzerland; 6 Roche Products Ltd, Welwyn Garden City, UK; 7 Patient Representative, Newcastle, Australia; 8 Instituto Nacional de Câncer, INCA, Rio de Janeiro, Brazil; a https://orcid.org/0000-0002-2470-6054

**Keywords:** clinical trials, regulatory approval, ethics committee/institutional review board, trial activation timelines

## Abstract

**Background:**

Geographic location and national income may influence access to innovation in healthcare. We aimed to study if geographical location and national income influenced the timelines to activate the global phase III APHINITY trial, evaluating adjuvant pertuzumab in patients with HER2-positive early breast cancer.

**Methods:**

Time from regulatory authority (RA) submission to approval (RAA), time to Ethics Committee/Institutional Review Board (EC/IRB) approval, time from study approval by EC/IRB to first randomised patient and from first to last randomised patient were collected. Analyses were conducted grouping countries by geographical region or economic income classification.

**Results:**

Forty-one countries (of 42) had data available regarding all relevant timelines. No statistical difference was observed between the time to RAA and geographical region (*p* = 0.47), although there was a trend to longer time to RAA in upper middle-income economies (*p* = 0.07). Except for time from first to last patient randomised, there was wide variation in timelines overall and within geographical regions and economic income groups.

**Conclusions:**

Geographical location and income classification did not appear to be the major drivers influencing time for clinical trial activation. Wide variability in activation timelines within geographical regions and income groups exists and is worthy of further investigation.

## Background

Clinical trials are essential for the development of new drugs, devices and diagnostic tests leading to patient benefit [1]. In cancer research, international collaborative clinical trials can significantly accelerate the recruitment of large patient populations usually required for phase III trials in order to generate scientific and clinical data in a timely manner [2]. Running clinical trials in geographically diverse patient populations also improves the generalisability of results across broader genomic, biological, ethnic and sociocultural backgrounds [3, 4]. Importantly, although disparities in access to clinical trials exist and inclusion of minorities in oncology clinical trials is beyond from optimal, clinical trials have the potential to improve patient care in developing countries, constructing local capacity to conduct clinical research and offering patients access to new treatments. This may play an important role in reducing the gap between the access of therapies in public and private health systems [5, 6].

The complexity of phase III clinical trials has, however, grown considerably and there are several administrative barriers that can contribute to increasing the length of time required to activate a clinical trial [2, 7–11]. This process depends on a complex network of multiple local and external oversight bodies with differing objectives and responsibilities [2, 9, 12]. Regulatory timelines are considered one of the most important elements in conducting clinical trials, and are seen by pharmaceutical companies as key indicators of a country’s attractiveness in relation to conducting a trial [5]. The European Union’s Clinical Trial Regulation EU 5362014, effective from 2019, calls for the avoidance of administrative delays for starting a clinical trial with a procedure that is ‘flexible and efficient without compromising patient safety or public health’ [13].

A previous analysis of regional timelines to initiate the Adjuvant Lapatinib and/or Trastuzumab Treatment Optimisation (ALTTO) global phase III trial (NCT00490139), which recruited patients between 2007 and 2011, suggested that geographical location and national income affect the time taken for trial initiation, being significantly longer in South American and upper middle-income economies; this potentially affects patient access to trials of innovative therapies in such locations [1].

To provide more recent data on this topic and to motivate a discussion around the activation process of phase III trials across the globe, we aimed to study if geographic location and income classification would influence the activation timelines of the phase III APHINITY trial (NCT01358877), which investigated the addition of pertuzumab to chemotherapy and Trastuzumab as adjuvant therapy for patients with HER2-positive early breast cancer.

## Methods

### Data source

APHINITY was a phase III global trial, designed by the Breast International Group in collaboration with a pharmaceutical industry sponsor, and was conducted under the auspices of an independent data and safety monitoring committee. The APHINITY trial activation timelines in each participating country were recorded by the pharmaceutical industry study sponsor and transferred to the study team for the purpose of this analysis. Data were compared across geographical regions (Europe, North America, Asia-Pacific, South America and Africa) and economic income groups (high, upper-middle and lower middle income) as defined by the World Bank [14, 15].

### Timelines

The following time intervals were evaluated for each participating country:

1. Time to regulatory authority (RA) approval, defined as the interval (in days) between protocol submission by the sponsor to the RA and its approval.
2. Time to Ethics Committee or Institutional Review Board (EC/IRB) approval, defined as the interval (in days) from submission by the study sponsor to EC/IRB and its approval.
3. Time from RA to first patient, defined as the interval (in days) between RA approval and first patient randomised into the study.
4. Time from EC/IRB to first patient, defined as the interval (in days) from first EC/IRB approval to the first patient included in the study.
5. Time from first to last patient, defined as the interval (in months) from the first patient randomised in the study in a particular country to the last patient randomised in the study (31 August 2013).

### Notes on deriving timelines

Protocol approval was not received from the national EC/IRB for Brazil in a timely fashion; Brazil did not, therefore, participate in the APHINITY trial. Additionally, the protocol was not submitted to the national RA for Israel so the corresponding timelines for Israel could not be calculated. The missing day in a partial date of protocol submission to country EC/IRB for Argentina and for Switzerland was imputed as day 15 as the middle of the month. Each individual patient’s date of randomisation was recorded in the randomisation system and uploaded to the APHINITY database.

### Geographical region classification

Each site’s country was classified according to World Bank guidelines [14] and grouped as follows: East Asia and Pacific, Europe and Central Asia, Latin America and Caribbean, North America, Middle East and North Africa, and sub-Saharan Africa (Supplementary Table 1).

### Economic income group classification

Economic income group was derived from each site’s country and based on the World Bank’s list of economies (June 2019) [15] and classified into the following economic income groups: lower-middle income, upper-middle income and high income (Supplementary Table 1); no low-income country participated in the APHINITY trial.

### Statistical analysis

Descriptive statistics, including means, medians and ranges, were calculated for the different timelines evaluated. Differences between geographical regions and economic income classification groups were calculated using one-way analysis of variance (ANOVA), following data normalisation on square roots of the different timelines obtained. Geographical regions represented by only one participating country were not included in the ANOVA calculations. ANOVA statistics was calculated only for time to RA approval as the process of EC/IRB approval does not follow a standard procedure being highly variable and influenced by local procedures, which could led to data misinterpretation. Differences were considered statistically significant if *p*-value was <0.05.

## Results

### Study demographics

APHINITY randomised 4,805 patients between November 2011 and August 2013 among 42 countries. Of the 42 countries, 21 (50.0%) were located in Europe and Central Asia, 9 (21.4%) in East Asia and Pacific, 8 (19.0%) in Latin America and Caribbean, 2 (4.8%) in North America, 1 in Middle East and North Africa (2.4%) and 1 (2.4%) in sub-Saharan Africa. Twenty-eight (66.7%) of the 42 countries had high, 11 (26.2%) upper-middle and 3 (7.1%) lower-middle income economies (Supplementary Table 1).

### Time to study activation across geographical regions

**Figure 1.**
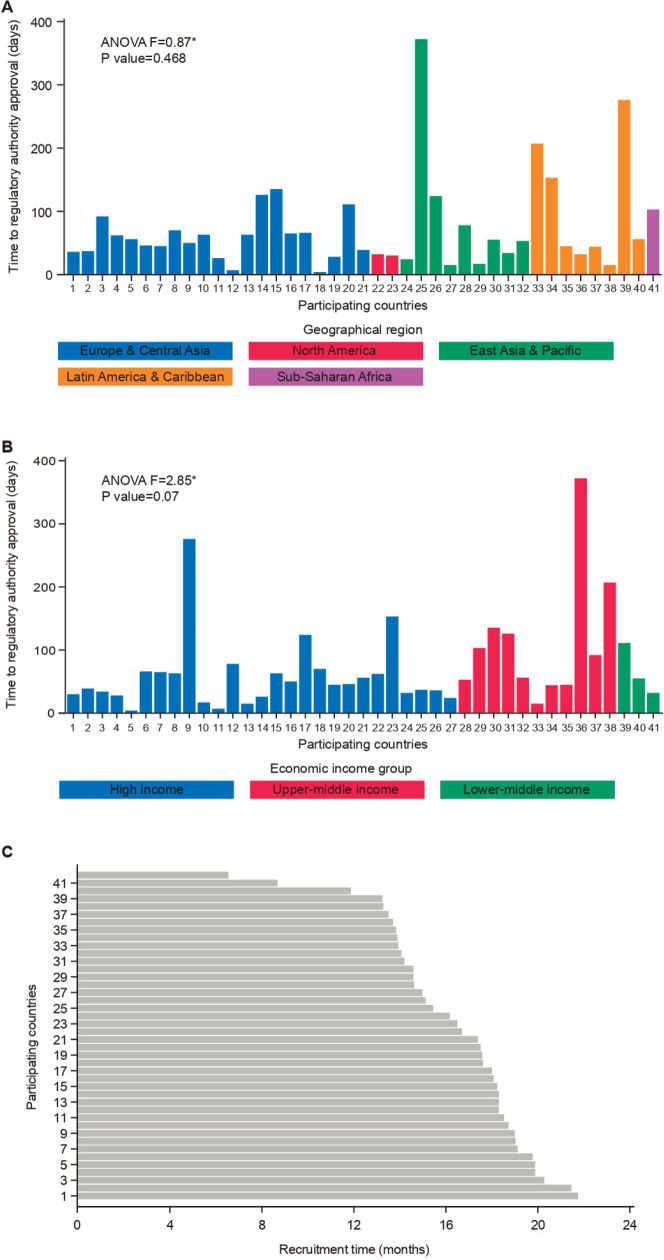
Timelines analysed in the APHINITY trial. A: Time to regulatory approval according to geographical region. B: Time to regulatory approval according to economic income group. C: Time interval from the first randomized patient in each country.

Table 1 summarises the timelines for study activation across geographical regions. Time to RA approval did not vary significantly across regions (ANOVA *F*: 0.87; *p* = 0.468; Figure 1a). North America had a numerically shorter time to RA approval (median: 31 days, range: 30–32) compared with Europe and Central Asia (median: 56 days, range: 4–135), East Asia and Pacific (median: 53 days, range: 15–372), Latin America and Caribbean (median: 51 days, range: 15–276) and sub-Saharan Africa (median: 103 days; range: 103–103). Regarding time to EC/IRB approval, a numerically longer time was observed for Middle East and North Africa (141 days; range: 141–141) when compared to the other geographic regions and overall EC/IRB submission to approval, which occurred with a median of 56 days (range: 14–421; Table 1).

Regarding the time interval between EC/IRB approval to first patient randomised, Latin America and Caribbean had a longer time interval (median: 232 days, range: 98–463) compared to other regions (overall time interval of 118 days, range: 13–463; Table 1).

Importantly, there was wide variation in timelines for trial activation overall and within geographical regions. For example, the median time for EC/IRB approval to first randomised patient across overall was 118 days, but the range was wide (range: 13–463) (Table 1). Further details on variation in time to RA approval in geographical regions represented by four or more countries are shown in Table 2.

### Time to study activation across economic income groups

The different time intervals for study activation across economic income groups are reported in Table 3. Although no statistically significant difference was found between economic regions regarding time to RA approval, there was a trend (ANOVA *F*: 2.85, *p* = 0.07; Figure 1b) to a longer time in upper-middle economies (92 days, range: 15–372 days) compared to the other groups (high income: 45 days, range: 4–372 days and lower-middle income: 55 days, range: 32–111 days; Table 3).

Similar to the findings for geographical regions, there was a wide variation in the timelines for study activation overall and within the economic income groups. For example, the median time for EC/IRB approval to first randomised patient across overall was 56 days, but again the range was wide (range: 14–421; Table 3). Further details on variation in time to RA approval in economic income groups represented by four or more countries are shown in Table 2.

### Recruitment period

The time intervals from the first randomised patient in each participating country to the last randomised patient in the study (study enrolment closure) were recorded for all participating countries and across the defined economic and geographic regions. This timeline was relatively homogenous between geographic regions (overall: 17 months, range: 6.5–21.7; Table 1) and economic regions (overall: 17 months, range: 6.5–21.7; Table 3). Figure 1c shows the time that elapsed between the first patient being randomised and study enrollment closure in each participating country. Of note, the recruitment period for APHINITY was short (less than 24 months).

## Discussion

Regulatory requirements have the aim of ensuring the safety and appropriate conduction of clinical trials, safeguarding the wellbeing of participants and providing assurance of research standards and data quality [12]. However, the high complexity of regulatory obligations related to clinical trials and their variability among different countries and regions of the world can increase the burden of documentation on investigators and the time taken to activate a clinical trial [2, 11].

Compared to the previous analysis of ALTTO [1], another phase III clinical trial that enrolled a similar population of patients with early-stage HER2-positive breast cancer, we observed an overall improvement in the timelines for study initiation across the globe. For example, the median time to RA approval in the Latin America and Caribbean regions in the APHINITY was 51 days (range: 15–276) compared to the 236 days (range: 21–257) for South America in the previous report [1]. An improvement in the time from EC/IRB approval to first patient randomised was also noted for all geographic regions in APHINITY compared to ALTTO (overall median time 118 versus 169 days) [1]. Caution is, however, needed in interpreting these results since the countries participating in ALTTO and APHINITY were not identical (Supplementary Table 2). In addition, we did not see a statistically significant difference between the time to RA approval according to geographical region or income classification in the APHINITY trial. This is to be welcomed and may reflect the collective work of collaborative research groups, pharmaceutical industry sponsors, contract research organisations and RA across the globe between the implementation of ALTTO in 2007 and APHINITY in 2011.

It is, however, important to note the wide variability in the timelines for trial initiation within geographical regions and income classification, suggesting that these two variables are not the main reasons for potential delays. Indeed, considering that all regulatory systems should share the same ultimate goals of protecting patients’ safety and assuring research standards, [12] country-specific differences may not correspond to disparities in regulatory needs, but more likely reflect differences in local operational procedures in individual sites and/or countries [2].

For example, an administrative challenge that can increase the time taken to initiate international trials in regions that speak different languages is the need for linguistic translation of patient informed consent forms and/or protocols [11]. This can be further prolonged if the local IRB or EC requires forward and back translation of trial documents [2, 4]. In addition, the time to trial initiation may be prolonged by the requirement to include specific institutional appendices or to edit the protocol according to specific institutional templates, fonts or styles [10] to satisfy local regulatory standards; such bureaucratic requirements do not necessarily translate into an improvement in patient experience, safety or research quality.

Another element that might contribute to the variability in timelines seen in our study is differences in drug supply and distribution between countries [2]. Distinct drug approval processes and healthcare systems across the globe lead to different importation and related requirements which can affect the ease of supply and access to treatment in different regions [2, 16]. In addition, variations in labelling, transport and storage requirements across different regions may also increase the length of study activation timelines and are also important to consider. Sometimes, clinical trials may require biospecimens for translational research and/or confirmation of trial eligibility but regulatory requirements related to the acquisition and management of biospecimens are heterogeneous across countries and may also affect activation timelines [2, 17, 18]. In some regions, it might be necessary to set up parallel biobanks or laboratories, which will need quality assurance processes to ensure reliability of the generated data [18, 19].

In an operational context, one means to reduce the time taken to activate clinical trial sites might be by conducting different activation steps in parallel instead of in series. ‘In tandem’ processing of financial, contractual and regulatory steps successfully reduced the time taken to activate clinical trials by 70% in three geographically diverse and distant Mayo Clinic sites [20]. The gains of this approach were achieved primarily by reducing non-value-added time (i.e., wait or rework time) between steps, reducing rework and eliminating unnecessary steps, rather than shortening the individual times required to conduct scientific and regulatory reviews or working longer hours [20].

In the European Union (EU), the Voluntary Harmonisation Procedure (VHP) is an initiative developed by the ‘Clinical Trials Facilitation Group’, a working group of the ‘Heads of Medicines Agencies’ [21]. The VHP was established in 2009 to foster simultaneous initiation of the authorisation procedure for clinical trials in more than one European member state by submitting a single application. The VHP has reduced the period required for the authorities to respond to the applicant of a multinational trial to a maximum 60 days in all EU countries involved [22]. During the period 2013–2018, 16%–23% of all multinational clinical trials in Europe underwent the VHP before submission to the national competent authorities [22]. Of note, during the APHINITY trial, the VHP process was not followed; therefore, it is not known whether the VHP could have impacted the results of our analysis.

We should, however, remember that study activation is only one of the facets where bureaucracy might hinder clinical cancer research. According to a survey of 940 clinical investigators, the number of administrative tasks related to clinical trials and their complexity have grown markedly during recent years, which they consider an obstacle to the development of clinical research [11]. Furthermore, there was a consensus about the potential to limit such procedures without compromising the safety and the rights of the patients or the quality of data [11]. Indeed, recently, several medical societies, as well as patient advocates, have called for urgent actions to diminish bureaucratic burdens and move towards more patient-centred, risk-based, pragmatic, efficient and cheaper trials [23]. The COVID-19 pandemic has given further impetus to these moves [24].

Some limitations of our research need to be acknowledged. First, the results from this analysis should be considered as exploratory, as they were not pre-planned; nevertheless, they warrant further evaluation in other phase III trials. In addition, although the geographic and or economic region were not identical in the two reports, 87.8% of the countries participating in APHINITY also participated in ALTTO, so there was considerable overlap. In our report, however, geographic and economic regions were classified according to the current World Bank Criteria (June 2019), while the prior ALTTO analysis only used World Bank Criteria to classify income groups and not geographic groups. In addition, as the results were presented by country (and region), there would have been wide variation in income of the populations of hospital sites, although these should not have affected nationwide procedures. Also, the rather small number of countries in some geographic regions, such as North America and Middle East and North Africa, as well as in the lower-middle income subgroup should be noted. Lastly, our findings may not apply to academic trials as they differ from industry sponsored trials in several aspects such as the resources available to address the regulatory, as well as financial and operational, obligations of international clinical trials.

## Conclusion

In conclusion, although general improvement in the timelines to activate a clinical trial was observed in the APHINITY trial compared to the previous ALTTO report, there was wide variability in the timelines within geographic and economic regions. Our results suggest that geographical regions and national income levels do not *per se* appear to be the major drivers behind a delay on activating a clinical trial. The causes of such delays should, therefore, be further investigated on a country and site-basis with a common interest in the safe and efficient delivery of clinical trials across the most diverse and representative patient populations possible.

## Ethics approval and consent to participate

The APHINITY trial (Breast International Group 01-06) is a randomised, multicenter, open-label, phase III study. The Ethics Committee and relevant health authorities at each participating institution approved the study protocol. All participants gave written informed consent before study entry.

## Consent for publication

This manuscript does not contain any individual person’s data in any form. Therefore, consent for publication is not applicable.

## Prior presentation

Partial results of this work have been presented as a poster at the 2020 San Antonio Breast Cancer Symposium (PS7-21).

## Data availability

Due to the Informed Consent Form, data privacy and Intellectual Property Rights-related restrictions, the clinical data cannot be made public, i.e., accessible for anyone, for any purpose without a review process and without putting an agreement in place. Nevertheless, raw data are available upon request and any request can be directed to the central APHINITY team.

## Conflicts of interest

MAF, OE, DP, SG and LR: none. CT: Travel grant, Roche; advisory board: AstraZeneca, Eisai, Daiichi Sankyo, MSD. Speaker: Pfizer and Eisai. MP: MP’s institution received funding from Roche in respect of the APHINITY trial. NP: advisory board: Lilly; contracted research: Novartis, AstraZeneca, Daichii Sankyo, Roche and Pfizer; speaker: Novartis, Lilly, AstraZeneca and Roche. DE: funding for his research fellowship (2018–2019): Novartis; speaker fee: Janssen; salary paid by Roche. EA: honoraria and/or advisory board from Roche/GNE, Novartis, Seattle Genetics, Zodiac and Libbs; travel grants from Roche/GNE and GSK/Novartis; research grant to my institution from Roche/GNE, Astra-Zeneca, GSK/Novartis and Servier. JB: travel expenses: AstraZeneca and Roche; consultant: AstraZeneca, Daiichi Sankyo, Genomic Health, Libbs, Lilly MSD, Novartis, Pfizer and Roche. EC: employee of Roche Products Ltd; issued patent: Uses for and article of manufacture including HER2 dimerisation inhibitor pertuzumab, 13/649591.

## Funding information

This sub-analysis has received no funding.

## Figures and Tables

**Supplementary Table 1.** Participating countries in the APHINITY trial classified by geographic region and income.

Country	Geographical region	Income classification
Argentina	Latin America and Caribbean	Upper middle income
Australia	East Asia and Pacific	High income
Austria	Europe and Central Asia	High income
Belgium	Europe and Central Asia	High income
Brazil	Latin America and Caribbean	Upper middle income
Bulgaria	Europe and Central Asia	Upper middle income
Canada	North America	High income
Chile	Latin America and Caribbean	High income
China	East Asia and Pacific	Upper middle income
Colombia	Latin America and Caribbean	Upper middle income
Croatia	Europe and Central Asia	High income
Czech Republic	Europe and Central Asia	High income
Denmark	Europe and Central Asia	High income
El Salvador	Latin America and Caribbean	Lower middle income
France	Europe and Central Asia	High income
Germany	Europe and Central Asia	High income
Guatemala	Latin America and Caribbean	Upper middle income
Hong Kong	East Asia and Pacific	High income
Hungary	Europe and Central Asia	High income
Ireland	Europe and Central Asia	High income
Israel	Middle East and North Africa	High income
Italy	Europe and Central Asia	High income
Japan	East Asia and Pacific	High income
Republic of Korea	East Asia and Pacific	High income
Mexico	Latin America and Caribbean	Upper middle income
Netherlands	Europe and Central Asia	High income
Panama	Latin America and Caribbean	High income
Peru	Latin America & Caribbean	Upper middle income
Philippines	East Asia and Pacific	Lower middle income
Poland	Europe and Central Asia	High income
Romania	Europe and Central Asia	Upper middle income
Russian Federation	Europe and Central Asia	Upper middle income
Slovenia	Europe and Central Asia	High income
South Africa	Sub-Saharan Africa	Upper middle income
Spain	Europe and Central Asia	High income
Sweden	Europe and Central Asia	High income
Switzerland	Europe and Central Asia	High income
Taiwan	East Asia and Pacific	High income
Thailand	East Asia and Pacific	Upper middle income
Ukraine	Europe and Central Asia	Lower middle income
United Kingdom	Europe and Central Asia	High income
United States	North America	High income

**Supplementary Table 2.** Participating countries in the APHINITY and ALTTO trials.

Countries	Participated in APHINITY	Participated in ALTTO
Argentina	✓	✓
Australia	✓	✓
Austria	✓	✓
Belgium	✓	✓
Brazil	✗	✓
Bulgaria	✓	✓
Canada	✓	✓
Chile	✓	✓
China	✓	✓
Colombia	✓	✗
Croatia	✓	✓
Czech Republic	✓	✓
Denmark	✓	✓
El Salvador	✓	✗
France	✓	✓
Germany	✓	✓
Greece	✗	✓
Guatemala	✓	✗
Hong Kong	✓	✓
Hungary	✓	✓
Ireland	✓	✓
Israel	✓	✓
Italy	✓	✓
Japan	✓	✓
Republic of Korea	✓	✓
Mexico	✓	✓
Netherlands	✓	✓
New Zealand	✓	✓
Norway	✗	✓
Pakistan	✗	✓
Panama	✓	✗
Peru	✓	✓
Philippines	✓	✓
Poland	✓	✓
Romania	✓	✓
Russian Federation	✓	✓
Singapore	✗	✓
Slovakia	✗	✓
Slovenia	✓	✓
South Africa	✓	✓
Spain	✓	✓
Sweden	✓	✗
Switzerland	✓	✓
Taiwan	✓	✓
Thailand	✓	✓
Ukraine	✓	✓
United Kingdom	✓	✓
United States	✓	✓

**Table 1.** Timelines in the activation process of the APHINITY trial across geographic regions.

Geographic region	Number of countries (%)	Time to RA approval (days) ^a^	Time to EC/IRB approval (days)	Time from EC/IRB approval to first patient (days)	Time from first patient to last patient randomised (months)
Europe and Central Asia	21 (50.0%)	56 (4–135)	67 (22–164)	109 (13–257)	17.6 (13.2–21.7)
North America	2 (4.8%)	31 (30–32)	73 (19–126)	126 (86–165)	17.6 (13.8–21.5)
East Asia and Pacific	9 (21.4%)	53 (15–372)	67 (31–421)	108 (56–147)	18.0 (8.7–19.9)
Latin America and Caribbean	8 (19.0%)	51 (15–276)	43 (19–273)	232 (98–463)	14.6 (6.5–17.5)
Middle East and North Africa	1 (2.4%)	-	141 (141–141)	92 (92–92)	13.9 (13.9–13.9)
Sub-Saharan Africa	1 (2.4%)	103 (103–103)	14 (14–14)	185 (185–185)	18.2 (18.2–18.2)
Overall	42 (100%)	53 (4–372)	56 (14–421)	118 (13–463)	17.0 (6.5–21.7)

Data are presented as median (range)

a The protocol was not submitted to a country RA for Israel. The corresponding timelines for Israel cannot be calculated

EC/IRB = Ethics Committee/Institutional Review Board; RA = regulatory authority

**Table 2.** Variation in the time to RA approval in APHINITY.

Geographic region/economic income group	Number of countries (%)	Median time to RA approval (days) ^a^	IQR for time to RA approval (days) ^a^
Europe and Central Asia	21 (50.0%)	56	29
East Asia and Pacific	9 (21.4%)	53	54
Latin America and Caribbean	8 (19.0%)	51	142
High income	28 (66.7%)	45	37
Upper middle income	11 (26.2%)	92	90
Overall	42 (100%)	53	46

a The protocol was not submitted to a country RA for Israel. The corresponding timelines for Israel cannot be calculated

Geographical regions and economic income groups represented by less than four countries are not described in the summary table

A median with a midpoint of 0.5 will be displayed rounded up to the nearest day

RA = regulatory authority; IQR = interquartile range

**Table 3.** Timelines in the activation process of the APHINITY trial across economic income groups.

Economic income group	Number of countries (%)	Time to RA approval (days) ^a^	Time to EC/IRB approval (days)	Time from EC/IRB approval to first patient (days)	Time from first patient to last patient randomised (months)
High income	28 (66.7%)	45 (4–276)	60 (19–273)	98 (13–257)	18.2 (11.9–21.7)
Upper middle income	11 (26.2%)	92 (15–372)	54 (14–421)	185 (73–463)	14.2 (6.5–18.2)
Lower middle income	3 (7.1%)	55 (32–111)	33 (32–78)	201 (147–209)	15.1 (13.5–17.4)
Overall	42 (100%)	53 (4–372)	56 (14–421)	118 (13–463)	17.0 (6.5–21.7)

Data are presented as median (range)

a The protocol was not submitted to a country RA for Israel. The corresponding timelines for Israel cannot be calculated

EC/IRB = Ethics Committee/Institutional Review Board; RA = regulatory authority

## References

[1] Metzger-Filho O, Azambuja E, Bradbury I (2013). Analysis of regional timelines to set up a global phase III clinical trial in breast cancer: the adjuvant lapatinib and/or trastuzumab treatment optimization experience. Oncologist.

[2] Tang M, Joensuu H, Simes RJ (2019). Challenges of international oncology trial collaboration – a call to action. Br J Cancer.

[3] Pocock S, Calvo G, Marrugat J (2013). International differences in treatment effect: do they really exist and why?. Eur Heart J.

[4] Vose JM, Chuk MK, Giles F (2017). Challenges in opening and enrolling patients in clinical trials. Am Soc Clin Oncol Educ Book.

[5] Barrios CH, Reinert T, Werutsky G (2018). Global breast cancer research: moving forward. Am Soc Clin Oncol Educ Book.

[6] Rolfo C, Caglevic C, Bretel D (2016). Cancer clinical research in Latin America: current situation and opportunities. Expert opinion from the first ESMO workshop on clinical trials, Lima, 2015: Table 1. ESMO Open.

[7] Lacombe D, Golfinopoulos V, Negrouk A (2020). Clinical research in Europe: who do we do all that for?. J Cancer Policy.

[8] Dilts DM, Cheng SK, Crites JS (2010). Phase III clinical trial development: a process of chutes and ladders. Clin Cancer Res.

[9] Dilts DM, Sandler AB, Baker M (2006). Processes to activate phase III clinical trials in a cooperative oncology group: the case of cancer and leukemia group B. JCO.

[10] Steensma DP, Kantarjian HM (2014). Impact of cancer research bureaucracy on innovation, costs, and patient care. JCO.

[11] Perez-Gracia JL, Awada A, Calvo E (2020). ESMO Clinical Research Observatory (ECRO): improving the efficiency of clinical research through rationalisation of bureaucracy. ESMO Open.

[12] European Medical Agency (EMA) Guideline for good clinical practice E6(R2). https://www.ema.europa.eu/en/documents/scientific-guideline/ich-e-6-r2-guideline-good-clinical-practice-step-5_en.pdf.

[13] Scavone C, di Mauro G, Pietropaolo M (2019). The European clinical trials regulation (No 536/2014): changes and challenges. Expert Rev Clin Pharmacol.

[14] The World Bank The world by region. https://datatopics.worldbank.org/sdgatlas/the-world-by-region.html.

[15] The World Bank World Bank country and lending groups. https://datahelpdesk.worldbank.org/knowledgebase/articles/906519-world-bank-country-and-lending-groups.

[16] Juan Bamberger RP Overcoming complexities of clinical trial supplies in Latin America. Appl Clin Trials.

[17] Hall JA, Daidone MG, Peters GJ (2011). Integrating collection of biospecimens in clinical trials: the approach of the European Organization for Research and Treatment of cancer. Biopreserv Biobank.

[18] Teodorovic I, Therasse P, Spatz A (2003). Human tissue research: EORTC recommendations on its practical consequences. Eur J Cancer.

[19] Hall JA, Brown R (2013). Developing translational research infrastructure and capabilities associated with cancer clinical trials. Expert Rev Mol Med.

[20] Watters JT, Pitzen JH, Sanders LJ (2018). Transforming the activation of clinical trials. Clin Pharmacol Ther.

[21] Krafft H, Bélorgey C, Szalay G (2012). Experience and further development with the voluntary harmonization procedure for multinational clinical trials in the European Union. Nat Rev Drug Discov.

[22] Heads of Medicines Agencies (HMA) Results of the voluntary harmonisation procedure 2009-2018. https://www.pei.de/SharedDocs/Downloads/EN/regulation-en/clinical-trials/results-vhp-procedure.pdf?__blob=publicationFile&v=2.

[23] Joint statement by medical societies and patient advocates: reducing bureaucracy in clinical trials: now is the time!. https://www.biomedeurope.org/images/news/2020/Coalition_statement_Reducing_bureaucracy_in_clinical_trials_240920.pdf.

[24] Nabhan C, Choueiri TK, Mato AR (2020). Rethinking clinical trials reform during the COVID-19 pandemic. JAMA Oncol.

